# Bastinado for Asphyxia

**Published:** 1888-02

**Authors:** Ida F. Norris

**Affiliations:** House Physician


### BASTINADO FOR ASPHYXIA.

EDITORS OF THE HOMŒOPATHIC PHYSICIAN: In the volume of *Transactions of the International Hahnemannian Association for* 1886, is a record of two cases of asphyxiation from Ether, treated successfully with the bastinado at the hands of Professor Carleton, after the common methods used for resuscitation had failed, including artificial respiration. You will be glad to know that the bastinado has saved another life. It came about in this way:

Yesterday, a patient in this house was etherized with care for operation. She seemed healthy; no lesion of the heart or other vital organ could be discovered; not more than quarter of a pound of ether was used, properly diluted with air, while one member of the staff watched the pulse and respiration, and did nothing else. The exact degree of anæsthesia desired was produced—that is, the conjunctiva became but slightly sensitive. Without warning, respiration quickly slacked up, followed soon after by loss of pulse, and all signs of life suddenly disappeared.

Without delay or confusion, the staff set about the work of restoration, which included hypodermic injections of brandy and artificial respiration. Then recollecting the bastinado, slapping the soles of the feet with slippers was added. Reaction did not follow. Professor Carleton was then hastily summoned from the lecture-room. Abruptly stopping his lecture to the students, he began to direct our movements. He thought our work was good, excepting that the respiratory movements were too rapid and not emphatic enough. That we corrected. Also that the bastinado was not applied with sufficient precision and force. As he attached much importance to this last-named procedure, he took the matter personally in hand. Holding a foot so that its sole was well exposed, he struck with a shoe, quickly repeated, heavy blows in the hollow of the foot. In a few seconds the patient scowled, drew up her leg slightly, and then sighed. He then served the other foot in like manner, which caused good reaction. Respiration and pulse soon became natural.

IDA F. NORRIS, M. D., *House Physician.*

